# Molecular dynamics investigations of mechanical behaviours in monocrystalline silicon due to nanoindentation at cryogenic temperatures and room temperature

**DOI:** 10.1038/srep16275

**Published:** 2015-11-05

**Authors:** Xiancheng Du, Hongwei Zhao, Lin Zhang, Yihan Yang, Hailong Xu, Haishuang Fu, Lijia Li

**Affiliations:** 1 School of Mechanical Science and Engineering, Jilin University, Renmin Street 5988, Changchun, Jilin 130025, China

## Abstract

Molecular dynamics simulations of nanoindentation tests on monocrystalline silicon (010) surface were conducted to investigate the mechanical properties and deformation mechanism from cryogenic temperature being 10 K to room temperature being 300 K. Furthermore, the load-displacement curves were obtained and the phase transformation was investigated at different temperatures. The results show that the phase transformation occurs both at cryogenic temperatures and at room temperature. By searching for the presence of the unique non-bonded fifth neighbour atom, the metastable phases (Si-III and Si-XII) with fourfold coordination could be distinguished from Si-I phase during the loading stage of nanoindentation process. The Si-II, Si-XIII, and amorphous phase were also found in the region beneath the indenter. Moreover, through the degree of alignment of the metastable phases along specific crystal orientation at different temperatures, it was found that the temperature had effect on the anisotropy of the monocrystalline silicon, and the simulation results indicate that the anisotropy of monocrystalline silicon is strengthened at low temperatures.

At present, cryogenic engineering attracts more and more attention due to its extensive application in different areas, for instance, nuclear fusion technology, superconductor science and technology, space exploration and so on. Under such extreme conditions, materials exhibit mechanical properties that differ from their properties at room temperature. Therefore, it is necessary to investigate the mechanical properties of materials at cryogenic temperatures to satisfy the demand of cryogenic engineering. Nanoindentation is one of the most effective methods to determine the mechanical properties of materials from micro- to nanoscale, including the hardness, Young’s modulus and creep performance^1^,^2^,^3^,^4^, etc.

In recent years, many researches based on indentation have been performed to study the characteristics of materials at cryogenic temperatures. For instance, Wei *et al.*^5^ carried out the research to investigate the strengthening mechanism of aluminum nitride (AIN) ceramics from 293 to 77 K. They found that the increase of fracture surface energy and elastic modulus could be conducive to the strengthening of AlN ceramics at cryogenic temperatures. Yi *et al.*^6^ studied the fracture behaviour of silicon carbide at cryogenic temperatures and found that both the hardness and fracture toughness increased with temperature decreasing. Zhang *et al.*^7^ examined the grain growth in nanocrystalline Cu under the microhardness indenter at ambient and cryogenic temperatures, and the results indicated that grain coarsening was even faster at cryogenic temperatures than at ambient temperature. Güçlü *et al.*^8^ carried out indentation test on YBCO polycrystalline superconductor at cryogenic temperatures, ranging from 50 to 293K, and found that the hardness and elastic modulus increased with temperature decreasing.

Monocrystalline silicon, as one of the most popular semiconductor materials, plays an important role in manufacturing of the micro-electro mechanical systems (MEMS), precision optics elements and electronic products. With the development of technology, it is very important to investigate the characteristics of monocrystalline silicon under extreme conditions. Over the past few decades, a variety of experimental^9^,^10^,^11^,^12^,^13^,^14^ and theoretical^15^,^16^,^17^,^18^,^19^ studies have been performed to investigate the mechanical properties and phase transformation of monocrystalline silicon. However, few studies investigated the effect of the temperature on the mechanical properties and phase transformation in silicon, and most of these studies were conducted at room temperature and elevated temperatures. For instance, Zhao *et al.*^4^ investigated the influence of the crystal orientation and the temperature on the hardness of monocrystalline silicon and found that the hardness of the silicon material decreases and the adhesiveness of silicon increases with temperature increasing. Singh *et al.*^20^ performed nanoindentation experiments on silicon to study the effect of the temperature on the metastable phases in silicon in the temperature range from 20 to 135 °C. Ruffell *et al.*^21^ studied the phase transformation of silicon induced by nanoindentation at elevated temperatures (25–150 °C). Khayyat *et al.*^22^ used Raman microscopy to investigate the phase transformation in silicon due to indentation at room temperature (300 K) and a cryogenic temperature of 77 K. However, because the metastable phase of silicon, Si-II (or *β*-Sn), is an intermediate phase which forms during the loading stage^23^ and disappears during the unloading stage, it is difficult to directly experimentally identify or detect the phase during or after the indentation tests process. Moreover, the temperature effect on the anisotropy of monocrystalline silicon has been little studied to date.

This study aimed to investigate the mechanical properties and identify the phases formed during nanoindentation in the temperature range from 10 to 300 K using molecular dynamics (MD) simulations. The mechanical properties of monocrystalline silicon were studied. The phase transformation mechanisms observed during loading and unloading were described and explained in detail. Additionally, the temperature effect on the anisotropy of monocrystalline silicon was discussed.

## Simulation Method

### MD simulation model

**Figure 1:**
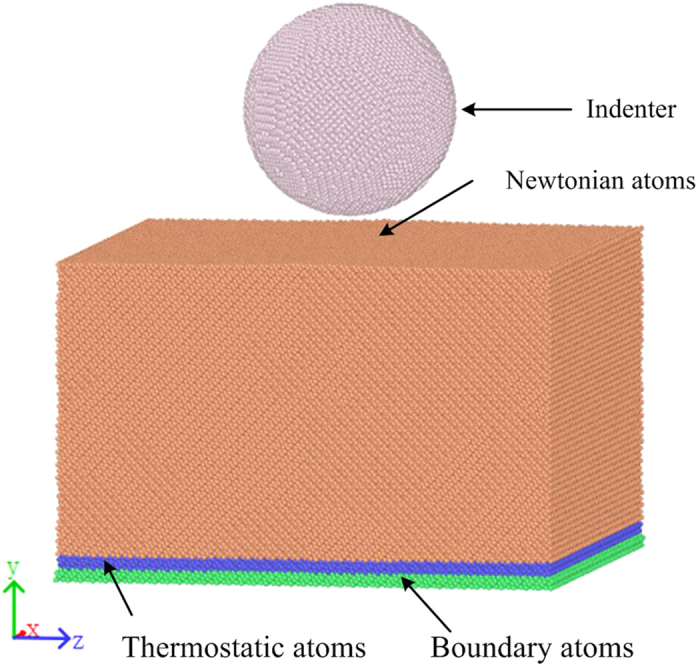
Three-dimensional MD model of the monocrystalline silicon and the nanoindenter.

For the simulation, a three-dimensional MD nanoindentation model was constructed, which is as illustrated in Fig. 1. The simulation model consists of a monocrystalline silicon substrate and a rigid spherical indenter with a radius of 5.0 nm. The model scale had to be large enough to eliminate the size effect, which would make the simulation computationally expensive^24^. Thus, a reasonable specimen volume was selected (21.7 nm × 13.57 nm × 21.7 nm), containing 325,240 atoms, based on the assumption that the velocities and displacements of the atoms during the nanoindentation simulation were not affected by the controlled volume. Periodic boundary conditions^4^,^18^,^24^,^25^ were chosen for the X and Z direction to reduce the effect of the simulation scale. The silicon atoms in the substrate were divided into three kinds of atoms, i.e., boundary atoms, thermostatic atoms and Newtonian atoms. The boundary atoms at the bottom of the substrate were fixed at their initial lattice position to provide structural stability and prevent the specimen from translating during the nanoindentation process. The next layers of atoms adjacent to the boundary atoms were considered to be thermostatic atoms to ensure a reasonable outward heat conduction. The remaining atoms were considered to be Newtonian atoms, i.e., their motion (as well as the motion of the thermostatic atoms) was set to obey Newton’s second law of motion.

### Potential energy functions selected for the MD simulations

The reliability of the simulation results depends on the accuracy of the MD simulation which is governed by the interaction potential function^16^. For covalent systems such as silicon, the effects of covalent bonds and the bond angle should be considered. The interatomic potential function proposed by Tersoff^26^ considers these effects and has been verified to be particularly feasible for modelling materials with a diamond cubic structure, e.g., carbon and silicon. Hence the Tersoff potential was adopted to simulate the interaction between the silicon atoms. The interaction between silicon and carbon atoms was modelled using the Morse potential according to literature, with *D* = 0.435 eV, *α* = 46.875 nm^−1^ and *r*_*0*_ = 0.19475 nm^23^.

### Initialization of the MD simulation

The simulation model was built assuming free boundary conditions in the Y direction, which is corresponding to the [010] direction of the silicon lattice. The indenter was positioned 10 Å above the centre of the surface of the silicon specimen at the beginning of the simulation and was assumed to move towards the substrate at a rate of 0.0005 Å per 1 fs. The MD model of the specimen was equilibrated to the target temperature, i.e., 10, 100, 200 or 300 K, respectively, assuming a microcanonical (NVE) ensemble. The initial velocities of the atoms were assigned in accordance with the Maxwell-Boltzmann distribution. The total kinetic energy was controlled to remain constant using the direct velocity scaling method to keep the temperature of the specimen close to the initial temperature.

The Large-scale Atomic/Molecular Massively Parallel Simulator (LAMMPS) MD program developed by Plimpton^27^ was used to perform the large scale three-dimensional MD simulations. Utilizing the Message Passing Interface (MPI) library, the parallel computation was realized.

## Results and Discussion

### Material properties at different temperatures

**Figure 2:**
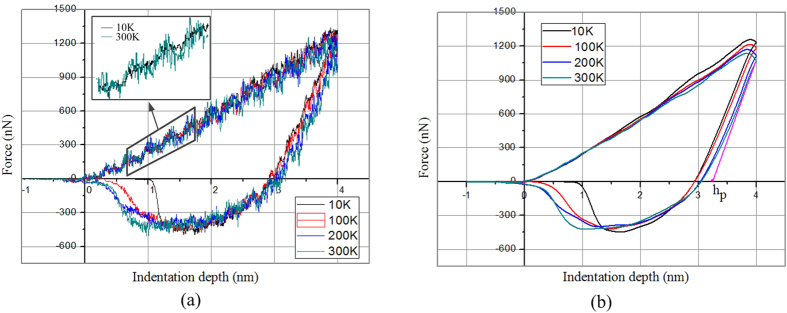
Load-displacement curves for nanoindentation at different temperatures. (**a**) Initial simulated curves; (b) Filtered curves.

Figure 2 shows the load-displacement (*P-h*) curves obtained for nanoindentation at cryogenic temperature of 10, 100 and 200 K, and room temperature (300 K). Figure 2a shows the simulated load-displacement curves and Fig. 2b shows the filtered load-displacement curves. In contrast to the macroindentation curve, the nanoindentation curve contains features a fluctuation due to the scale of the simulation, as shown in Fig. 2a. The fluctuation increases with temperature increasing. This is attributed to the stronger vibration of the atoms in the interaction area between the indenter and the silicon substrate at higher temperatures. Adhesive contact occurs as the load value becomes negative, due to the attraction between the substrate atoms and the indenter atoms. However, the period of adhesive interaction decreases with decreasing temperature.

The plastic indentation depth, *h*_*p*_ (=*h*_*max*_ *– P*_*max*_*/S*_*max*_)^28^, is defined as the intersection of the tangential line of the unloading curve with the *x*-axis, where *h*_*max*_ is the maximum indentation depth, *P*_*max*_ is the corresponding maximum load and *S*_*max*_ is the slope of the unloading curve at the maximum load. As shown in Fig. 2b, the plastic indentation depth decreases with decreasing temperature, which means that the elastic recovery is larger at a lower temperature. From a microscopic perspective, this might be attributed to the decreasing interatomic distance as a result of the lower temperature, leading to a stronger interaction between the atoms in the substrate. This is in agreement with the observation that the load at 10 K is highest during the early stage of the unloading process.

Quantitative information such as the hardness can be obtained from the *P-h* curve. The nanoindentation hardness *H* can be defined as follows:


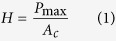


where *P*_*max*_ is the maximum indentation load, and *A*_*c*_ is the projected contact area calculated using the following equation:


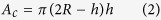


where *R* is the radius of the indenter and *h* is the indentation depth.

Then, the hardness of the monocrystalline silicon specimen was calculated using equations (1) and (2) to 17.4, 16.5, 15.8 and 15.1 GPa at temperature of 10, 100, 200 and 300 K, respectively. It can be easily seen that the hardness decreases with the temperature increasing. From a microscopic perspective, this behaviour can be explained as follows: the interatomic distance increases with the temperature increasing, leading to a decrease of the bond energy of the atoms in the substrate, which results in the decline of the hardness.

### Phase transformation during the nanoindentation process

**Figure 3:**
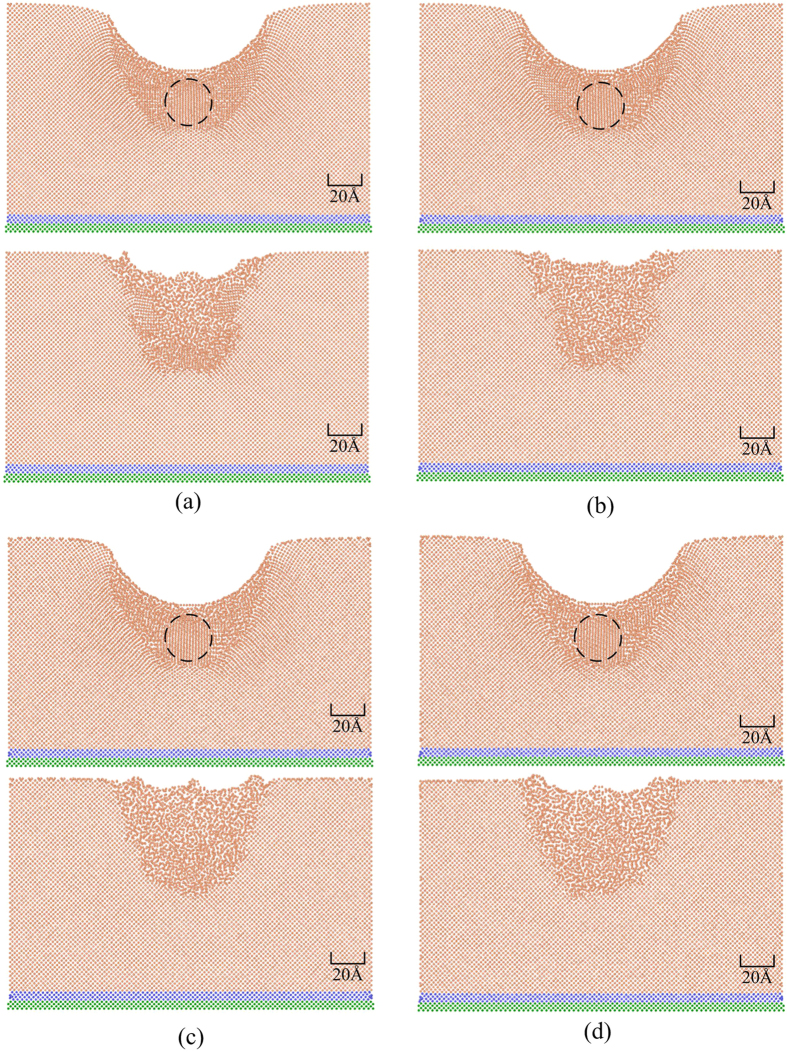
Deformation behaviour of the specimen at the maximum indentation depth and after unloading at different temperatures: (**a**) 10 K, (**b**) 100 K, (**c**) 200 K, (**d**) 300 K.

Figure 3 shows the positions of the atoms in the silicon specimen during the nanoindentation process at the two different temperatures. In order to more clearly observe the changes in crystalline order, the size of the silicon particles has been deliberately reduced. It can be observed that the transformed atoms beneath the indenter (marked by the dark circle) maintain a long-range crystalline order during loading. However, the atomic order of the transformed region is different compared with the original pattern, suggesting that a phase transformation occurred from one crystalline structure of silicon to another. In addition, the phase transformations are different at different temperatures, which will be discussed in detail in the following sections. During unloading, the atoms in this region lose its long-range crystalline order.

It is well known that a phase transformation from the diamond cubic structure (Si-I) to the *β*-tin structure (Si-II) occurs when the hydrostatic pressure exceeds a certain threshold. The threshold pressure required to induce this phase transformation was experimentally determined to 12.0 GPa^9^. The phase transformation can also occur at a lower compressive pressure in the presence of shear stress. Because the hydrostatic pressure is associated with a volume change leading to the phase transformation^29^, it is important to determine the pressure distribution in the substrate to decide whether a phase transformation to Si-II has occurred during the nanoindentation process.

**Figure 4:**
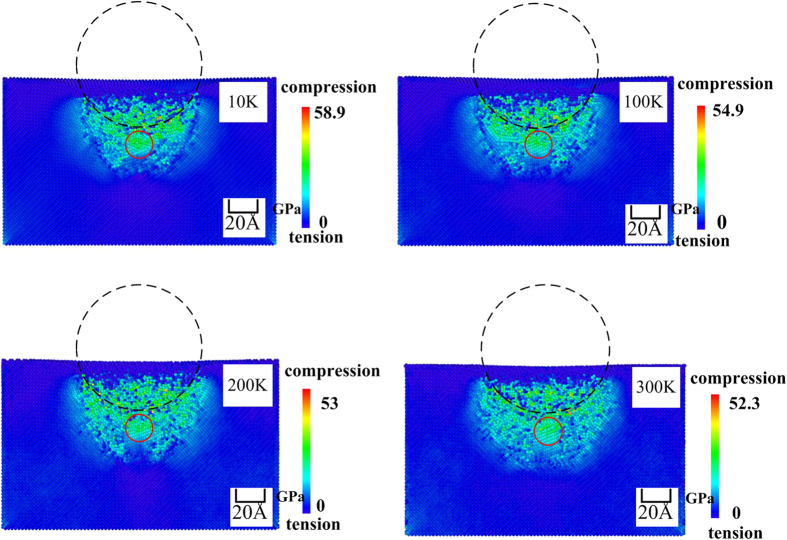
Lateral cross-sectional illustration of the hydrostatic stress at the maximum indentation depth at different temperatures.

The hydrostatic stress was calculated using the following equation:


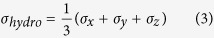


where ***σ***_***x***_ , ***σ***_***y , ***_***σ***_***z***_ are the normal stresses in the X, Y, and Z direction, respectively, and are calculated using the virial theorem^30^. The hydrostatic stress was computed by dividing the simulation space into 5.43 × 5.43 × 5.43 Å^3^ cells^31^, where 5.43 is the lattice distance. The atomic volume of the silicon was assumed as the cell volume divided by the number of atoms in a given cell^19^. The per-atom volume computed this way may be slightly larger than the actual volume because the substrate surface is compressed by the indenter^20^. This means that the calculated stress (pressure) is slightly too low for some atoms. However, this will not affect the decision whether the pressure has exceeded the threshold for inducing the phase transformation. Figure 4 shows an illustration of the lateral cross-sectional distribution of the hydrostatic stress in the specimen at the maximum indentation depth at the different temperatures. As shown in Fig. 4, the hydrostatic stress beneath the indenter is larger than 12 GPa. Therefore, we can assume that a phase transformation from Si-I to Si-II has occurred at the four different temperatures.

There are several possible phase transformation mechanisms in silicon, and it is generally accepted that Si-I transforms to Si-II during the loading stage. The presence of the Si-I, Si-II, Si-III, Si-XII and bct5-Si phases has been experimentally observed and discussed by Boyer *et al.*^32^ Generally, the crystal structures can be classified using radial distribution function (RDF) in MD simulations. However, RDF is not useful for identifying a specific structure when different phases are mixed together in a small volume. To identify the different structural phases formed in the transformed region, in this work, the surrounding environment of an atom with a radius larger than the maximum bond length was considered. The surrounding environment of each atom was divided into two sections: the range of the first section was reached to the maximum bond length and the range of the second section was set to include non-bonded second neighbours. The number of neighbour atoms in each sectional area was used for identification. At atmospheric pressure, the Si-I phase exhibits a diamond cubic structure with each atom having four nearest neighbours at a distance of 2.35 Å. The atoms in the two metastable phases (Si-III and Si-XII) also have four nearest neighbours at a distance of 2.37 Å and 2.39 Å^12^, respectively. Thus, these two metastable phases can be hardly distinguished from the Si-I phase in the MD simulation studies just using the coordination number. However, a unique non-bonded fifth neighbour can be used to distinguish these metastable phases from the Si-I phase^17^. According to literature^12^,^33^,^34^, at atmospheric pressure, the Si-I phase atoms have 12 non-bonded second neighbours at a distance of 3.83 Å, whereas the Si-III and Si-XII phase atoms have a unique neighbour at a distance of 3.41 Å and 3.23 Å (or 3.36 Å), respectively, at a pressure of 2 GPa. Because the maximum bond length is lower than 2.6 Å, the fourfold coordinated atoms can be as assumed to belong to the two metastable phases if they have non-bonded neighbours in the range from 2.6 to 3.5 Å^17^.

**Figure 5:**
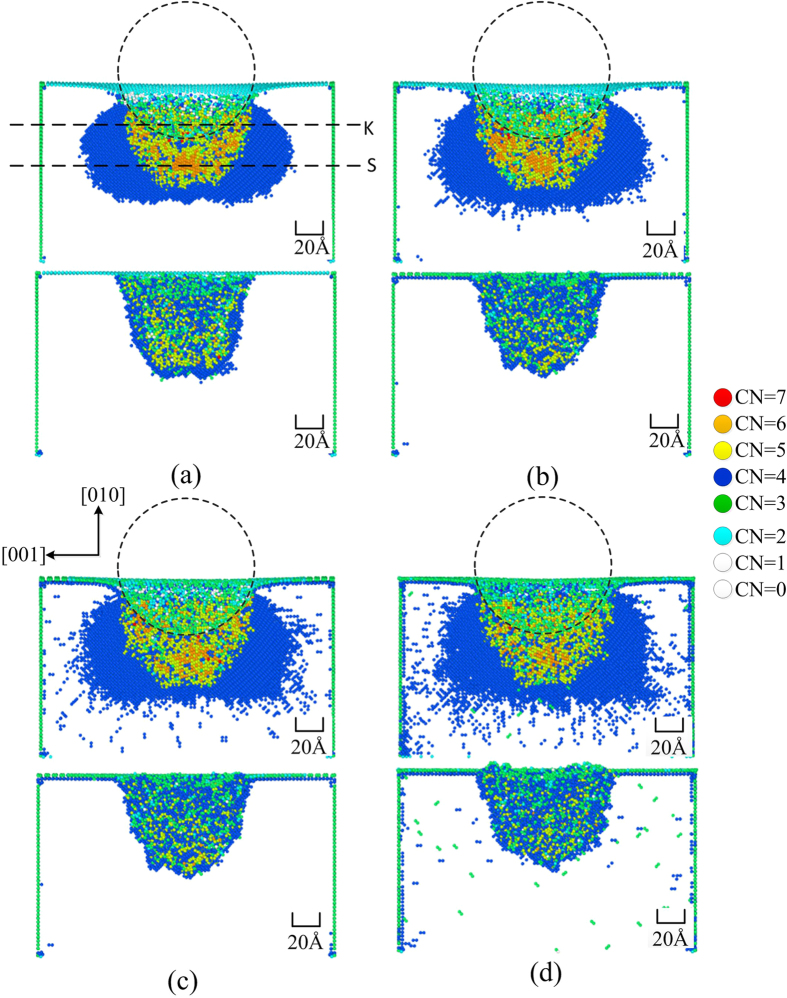
Lateral cross-sectional view of the transformed region at the maximum indentation depth and after unloading at different temperatures: (**a**) 10 K, (**b**) 100 K, (**c**) 200 K, (**d**) 300 K. The cross-sectional view on the (100) plane is passing through the centre of the simulation model (plane T in Fig. 7).

Figure 5 shows a lateral cross sectional view of the deformed silicon specimen at different stages of the nanoindentation at different temperatures. The silicon atoms of the specimen are coloured according to their coordination number. The Si-I phase with fourfold coordination is not shown in the diagram. In Fig. 5, it can be seen that the six-coordinated Si-II phase is located in the region beneath the indenter at the maximum penetration depth. Some other six-coordinated atoms are embedded in the five-coordinated atom sea. The two metastable phases, Si-III and Si-XII, are observed around the region containing atoms with fivefold and sixfold coordination. It can be easily seen that the two metastable phases are distributed more regularly at lower temperatures. After unloading, most of the Si-III and Si-XII atoms are transformed to Si-I, while the Si-II phase transforms into the amorphous phase, indicating that the phase transformation from Si-I to Si-III and Si-XII during loading is reversed during unloading, whereas the phase transformation from Si-I to Si-II is irreversible. The amorphous phase is formed in the residual transformed region, containing fourfold, fivefold and sixfold coordination atoms. Since the metastable phases transform back into their original structure, we propose that this transformation is related to the elastic recovery of silicon during the retraction process.

**Figure 6:**
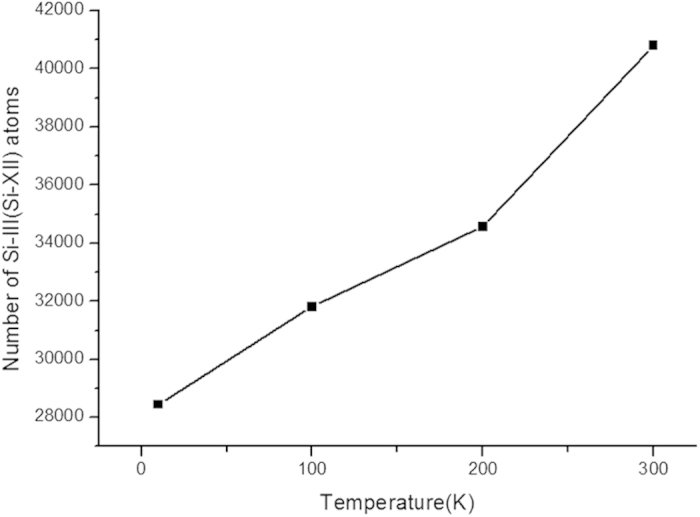
Number of Si-III and Si-XII atoms at the maximum indentation depth at different temperatures.

Figure 6 shows the number of Si-III and Si-XII atoms at the maximum penetration depth at different temperatures. The number of Si-III and Si-XII atoms increases with the temperature, indicating that the two metastable phases are more easily formed at higher temperatures.

**Figure 7:**
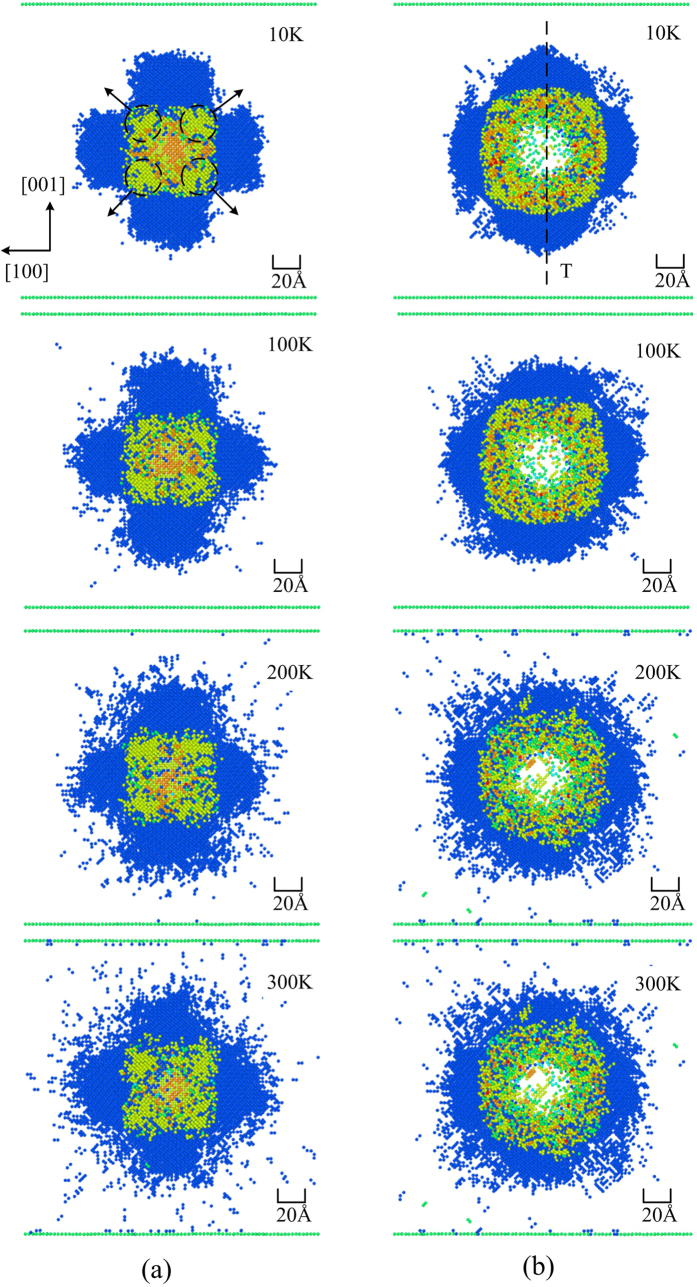
Horizontal cross-sectional view of the transformed regions at different depths from the Si (010) surface at a temperature of 10, 100, 200 and 300 K, respectively. (**a**) At a depth of 6–7 nm from the surface (plane S in Fig. 5); (**b**) 3–4 nm from the surface (plane K in Fig. 5).

The temperature was found to affect both the phase distribution and the phase transformation. To better understand the phase distribution at different temperatures, the transformed regions were studied using cross-sectional views at different depths from the Si (010) surface, as shown in Fig. 7. We can see that the five-coordinated structure is aligned along the <110> family of directions, namely 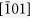, 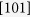, 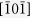 and 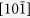, at all four temperatures. The amorphous phase is distributed around the contact surface between the indenter and the substrate. Although the metastable phases, i.e., Si-III and Si-XII, generally occurred during the unloading process^12^,^14^,^20^,^21^, in this work, these phases were observed between the directions of <110> during the loading process. The presence of Si-III and Si-XII during loading was first confirmed by Kim *et al.*^17^. The metastable phases, Si-III and Si-XII, are aligned along special crystal orientations, namely, [100] and [001], but not along all directions, which is attributed to the anisotropy of monocrystalline silicon. However, with increasing temperature, the degree of alignment of the two metastable phases along the specific crystal orientation is reduced, which indicates that the degree of anisotropy of monocrystalline silicon decreases with the temperature increasing. It is worth noting that the effect of the temperature on the anisotropy of monocrystalline silicon is similar to the effect of the temperature on the anisotropy of stress-rupture observed for the nickel-based single-crystalline superalloy SRR99 studied by Han *et al.*^35^.

**Figure 8:**
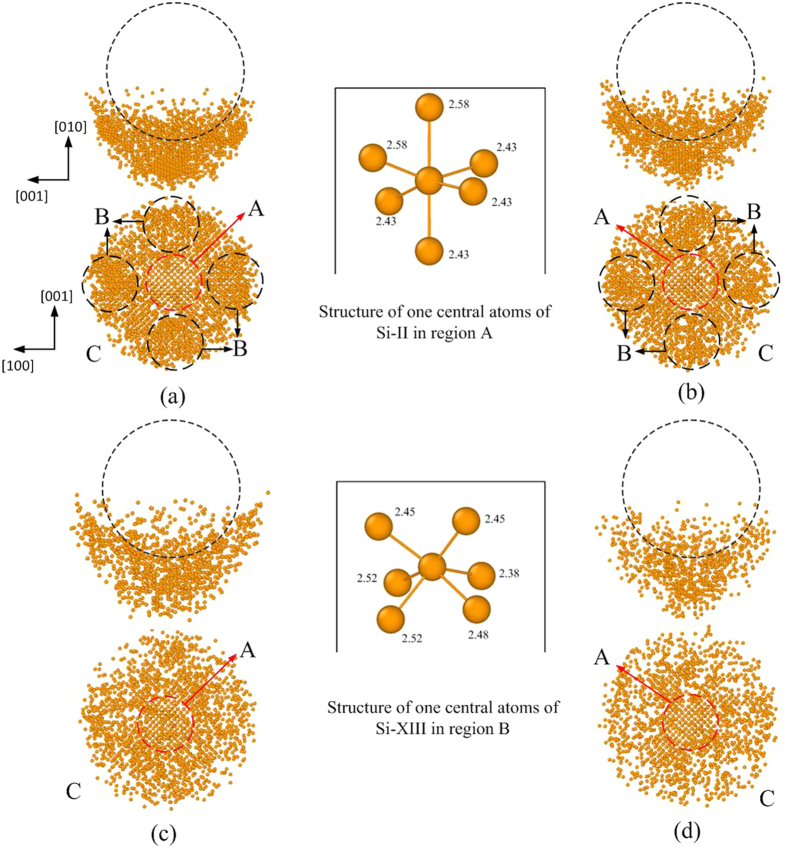
Lateral and horizontal cross-sectional view of the distribution of the six-coordinated atoms at the maximum indentation depth at different temperatures: (**a**) 10 K, (**b**) 100 K, (**c**) 200 K, (**d**) 300 K.

To analyze the phase transformation from the four-coordinated structure to the six-coordinated structure at different temperatures in more detail, lateral and horizontal cross-sectional views of the distribution of the six-coordinated atoms at the maximum indentation depth are shown in Fig. 8. The phase transformation and phase distribution are different at different temperatures. At a temperature of 10 and 100 K, the Si-II phase is formed beneath the indenter in region A (marked with a red circle). The Si-XIII phase, which was firstly found and defined by Mylvaganam *et al.*^36^ via MD simulation, is located in region B (marked with a dark circle), aligned along the [100] and [001] direction. Both the Si-II and the Si-XIII phase have six-coordinated atoms. However, the distance to the nearest neighbours is different^36^, as shown in the enlarged view in Fig. 8. The amorphous phase with six-coordinated atoms is formed between the nearest regions filled with the Si-XIII phase. At a temperature of 200 and 300 K, only the Si-II phase and the amorphous phase are formed, and a phase transformation from Si-I to Si-XIII does not occur. The atoms of the six-coordinated amorphous phase (in region C) are distributed around the Si-II phase.

**Figure 9:**
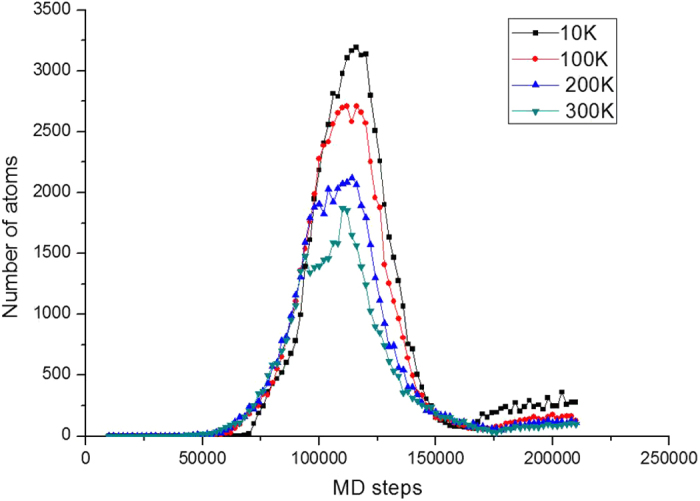
Number of atoms with the specified nearest number of neighbours during the nanoindentation process at different temperatures.

Figure 9 shows the variation of the number of six-coordinated atoms during the nanoindentation process at the different temperatures. No phase transformation occurs during the initial 10,000 MD steps. As the indenter penetrates the substrate, a fraction of the silicon atoms is transformed from the initial four-coordinated diamond cubic structure to a six-coordinated body-centred tetragonal structure. The number of transformed atoms is higher for nanoindentation at lower temperatures. The maximum number of transformed atoms at a temperature of 10 K is about twice the number observed at a temperature of 300 K. In addition, the difference in the maximum number of transformed atoms is higher in the interval from 100 to 200 K compared with the interval from 200 to 300 K, mainly because of the appearance of the Si-XIII phase atoms during nanoindentation, suggesting that the appearance of Si-XIII atoms facilitates the phase transformation process of monocrystalline silicon during nanoindentation. During unloading, the number of six-coordinated silicon atoms rapidly decreases, and only a few transformed atoms are distributed in the residual deformed region after the indentation.

## Conclusions

Molecular dynamics simulations were employed to simulate nanoindentation tests on a monocrystalline silicon (010) surface in the temperature range from ultra-low temperatures to room temperature. The effect of the temperature on the mechanical properties and phase transformation mechanisms was investigated. The main conclusions can be summarized as follows:

1. The load-displacement curves were simulated for different temperatures. The plastic indentation depth increases with the temperature increasing, which means that the elastic recovery is larger at a lower temperature. The hardness was obtained from the load-displacement curve and found to decrease with the temperature increasing.
2. During the nanoindentation process, phase transformation occurs both at low temperatures and at room temperature. The fourfold coordinated metastable phases, Si-III and Si-XII, were distinguished from the Si-I phase and were observed between the directions of <110>. Moreover, the temperature was found to affect the anisotropy of monocrystalline silicon: the degree of anisotropy decreased with the temperature increasing.
3. The phase transformation from a four-coordinated structure to a six-coordinated structure also depends on the temperature. At low temperatures (10 and 100 K), the Si-I phase is transformed to Si-II and Si-XIII during the loading stage, whereas no Si-XIII phase is formed at 200 K and at room temperature (300 K).

Moreover, in the present work, we have reported the mechanical behaviours of monocrystalline silicon during nanoindentation process at low temperatures from an atomistic point of view. Further analysis based on experiments would be done in our further research work.

## Additional Information

**How to cite this article**: Du, X. *et al.* Molecular dynamics investigations of mechanical behaviours in monocrystalline silicon due to nanoindentation at cryogenic temperatures and room temperature. *Sci. Rep.* **5**, 16275; doi: 10.1038/srep16275 (2015).
